# Natural populations of *Arabidopsis thaliana* differ in seedling responses to high-temperature stress

**DOI:** 10.1093/aobpla/plv101

**Published:** 2015-08-18

**Authors:** Nana Zhang, Brian Belsterling, Jesse Raszewski, Stephen J. Tonsor

**Affiliations:** 1 Department of Biological Sciences, University of Pittsburgh, 4249 Fifth Ave., Pittsburgh, PA 15260, USA; 2 Carnegie Museum of Natural History, 4400 Forbes Ave., Pittsburgh, PA 15213, USA

**Keywords:** Adaptation, *Arabidopsis*, climates, Hsp101, natural variation, thermotolerance

## Abstract

Little is known about adaptive within-species variation in thermotolerance in wild plants despite its likely role in both functional adaptation at range limits and in predicting response to climate change. Heat shock protein Hsp101, rapidly heat induced in *Arabidopsis thaliana*, plays a central role in thermotolerance in laboratory studies, yet little is known about variation in its expression in natural populations. We explored variation in thermotolerance and Hsp101 expression in seedlings from 16 natural populations of *A. thaliana* sampled along an elevation and climate gradient. We tested both naive controls (maintained at 22 °C until heat stress) and thermally pre-acclimated plants (exposed to a 38 °C 3-h acclimation treatment). After acclimation, seedlings were exposed to one of two heat stresses: 42 or 45 °C. Thermotolerance was measured as post-stress seedling survival and root growth. When stressed at 45 °C, both thermotolerance and Hsp101 expression were significantly increased by pre-acclimation. However, thermotolerance did not differ between pre-acclimation and control when followed by a 42 °C stress. Immediately after heat stress, pre-acclimated seedlings contained significantly more Hsp101 than control seedlings. At 45 °C, Hsp101 expression was positively associated with survival (*r*^2^ = 0.37) and post-stress root growth (*r*^2^ = 0.15). Importantly, seedling survival, post-stress root growth at 45 °C and Hsp101 expression at 42 °C were significantly correlated with the home sites' first principal component of climate variation. This climate gradient mainly reflects a temperature and precipitation gradient. Thus, the extent of Hsp101 expression modulation and thermotolerance appear to be interrelated and to evolve adaptively in natural populations of *A. thaliana*.

## Abstract (precis)

Heat stress limits species distribution, especially under predicted global climate change. The research article focuses on identifying the adaptive variation in response to high-temperature stress across an elevation gradient in natural *Arabidopsis thaliana* populations. We show that the accumulation of Hsp101, an important heat shock protein known to be essential for acquired thermotolerance, was positively associated with seedling survival and post-stress root growth. Pre-acclimation significantly increased thermotolerance at 45°C but not 42°C. Both Hsp101 and thermotolerance were correlated with the climate variation of home sites. Our study contributes to growing knowledge on abiotic stress responses in natural plant populations.

## Introduction

Increasing temperatures in many regions of the world may be a defining environmental change in the 21st century. Increasing heat stress is highly likely to lead to shifts in geographic distributions or even to complete extinction of many species (Field *et al*. 2014). Even without current human-driven rapid changes in thermal environments, populations at the warmer edge of their species' geographic range often face strong stresses from high-temperature events (Gaston 2009). Variation in thermal environment among sites within species' ranges may result in adaptive variation in thermotolerance. However, little is known about the within-species ability to evolve thermotolerance responses (Barua *et al*. 2008; Tonsor *et al*. 2008; Paul *et al*. 2011). In this study, we examine variation among natural populations of *Arabidopsis thaliana* in their ability to survive and recover from heat stress. We also test the extent to which survival and recovery from heat stress is correlated with the expression of heat shock protein (Hsp).

Exposure to high temperature can cause severe, irreversible cellular damage and loss of cellular function (Shabala 2012). The threshold temperature at which irreversible damage begins can vary among populations from contrasting in climates (Barua *et al*. 2008). The threshold temperature for damage can also be plastic in response to conditions experienced prior to high-temperature exposure (Wehner *et al*. 1985). When temperatures increase gradually or when plants experience a prior exposure to moderately high temperatures, changes in gene expression ensue (Larkindale *et al*. 2005; Hannah *et al*. 2006; Larkindale and Vierling 2008), leading to greater thermotolerance. This reprogramming and subsequent increase in thermotolerance is termed acclimation or acquired thermotolerance.

Acquired thermotolerance has been shown to depend, in part, on the rapid expression of Hsps (Lindquist 1986; Wang *et al*. 2004). Heat causes protein denaturation, disrupting normal protein function. The Hsps act as molecular chaperons to prevent protein aggregation, repair protein damage and maintain cellular homeostasis. Hsps stabilize protein form and prevent aggregation. The Hsps together recover or protect normal cellular function provided that the heat stress is not too extreme (Hong and Vierling 2001). While some other Hsps have been shown to have important roles in the heat shock response and in thermotolerance, to date only Hsp101 has been shown to be essential for acquired thermotolerance in plants. Hsp101 has a specific role as a protein machine that disaggregates misfolded proteins (Doyle and Wickner 2009).

This has been demonstrated in both *A. thaliana* (Hong and Vierling 2001) and maize (Nieto-Sotelo *et al*. 2002). Hsp101, the cytosol-expressed homologue of the Hsp100/ClpB gene family in *A. thaliana*, re-solubilizes and refolds denatured protein. Hsp101 is present in a single copy, Athsp101, in the *A. thaliana* genome. To date, nearly all plant studies of Hsp100/ClpB gene products have focussed on understanding Hsp101 genetics and biochemistry using genetic derivatives of the most commonly studied genotype of *A. thaliana*, Columbia (Col-0), in laboratory settings (Larkindale and Knight 2002; Hong *et al*. 2003; Larkindale *et al*. 2005; Tonsor *et al*. 2008).

Only a little is known about the extent to which natural populations evolve different patterns of Hsp101 expression in response to the differing patterns of heat stress they have experienced in nature. Natural populations of wild species can be important tools for studying the evolution of thermotolerance because they can carry adaptive signatures of selection imposed by their climates of origin. We know of only four studies that directly investigate the relationship between Hsps and thermotolerance in natural plant populations. Barua *et al*. (2003) demonstrated variation among ecotypes of *Chenopodium album* in thermotolerance of photosynthetic electron transport. This thermotolerance variation was strongly associated with variation in chloroplast small Hsp expression and with the thermal environments from which the genotypes were collected (Barua *et al*. 2003). Barua *et al*. (2008) further characterized geographically based variation among genotypes of *C. album* in Hsp60, Hsp70 and small Hsp expression, a finding that expression was greatest in genotypes from habitats that were subject to the greatest fluctuations in temperature. Habitats with high mean and maximum temperatures did not show greater Hsp expression (Barua *et al*. 2008). Similarly, Tonsor *et al*. (2008) tested standard accessions of *A. thaliana* whose sites of origin came from a broad latitudinal range. These accessions showed co-variation between latitude of origin and leaf Hsp101 content, with genotypes from warmer latitudes exhibiting lower content (Tonsor *et al*. 2008). Amano *et al*. (2012) compared two species of *Potamogeton* (Potamogetonaceae); one species was heat tolerant, and the other was heat intolerant. *Potamogeton malainus* from shallow waters with high-temperature fluctuations showed higher thermotolerance, while *P. perfoliatus* from deeper waters with cooler, more constant temperatures had lower basal thermotolerance and could not develop acquired thermotolerance because of changes in some of the heat shock element binding sites for HSFA2 (Amano *et al*. 2012). Taken together, these studies suggest that environments that differ in temperature regimes may lead, in general, to evolved differences in heat shock responses. The studies further suggest that evolved responses are likely the result of complex interactions of plants with their local climates. Predictions as to the expected pattern of response are, therefore, not likely to be accurate. Instead, we chose to simply ask: what are the extant patterns of responses?

Population-level studies exploring the relationship among the climate of origin, differentiation in thermotolerance and the role of changes in gene expression in acquired thermotolerance are needed to improve understanding of optimal heat stress responses in plants. Studies at the population level can provide insights on how populations, from common genetic background, adapted in the past to the novel climates in which they found themselves. Such studies can also help us predict more finely on how plants will respond to future climate shifts. Evolution of Hsps and heat shock response at large geographic scale has been largely conducted in animal species (Feder 1999). In fruit flies, *Drosophila virilis* from low latitude showed higher thermotolerance and Hsp70 accumulation after 40–41 °C heat shock (Garbuz *et al*. 2003). In a marine system, differential response to heat stress in invasive vs. native blue mussels showed the importance of heat shock response to invasive success (Lockwood *et al*. 2010). Heat shock proteins were found to have different roles in heat stress response, but to have generally been up-regulated (Sørensen *et al*. 2001, 2005; but see Jensen *et al*. 2010). Here, we identify adaptively differing patterns of Hsp expression that correspond with adaptation to contrasting thermal climates in the genomically highly enabled study system, *A. thaliana*. These results provide fundamental insights needed for future studies of the genetics, biochemistry and evolutionary ecology of variation in Hsp expression and thermotolerance in natural populations.

Many climate variables are correlated with elevation (Körner 2007), and elevation gradients create climate gradients across short distances that substantially mimic the broader gradients incorporated in many species' full geographic ranges. Thus, studying plants along an elevation gradient can simplify the logistics of climate–plant adaptation studies (Montesinos-Navarro *et al*. 2011). With F. Xavier Picó (La Doñana Biological Station, Seville, Spain), we collected 16 natural populations of *A. thaliana* along a climate and elevation gradient in southern Europe extending from near sea level at the Mediterranean coast to near treeline (∼2200 m above sea level (a.s.l.)) in the Pyrenee Mountains. Low-elevation sites are hotter and dryer overall, while high-elevation sites are cooler and wetter (Montesinos-Navarro *et al*. 2009, 2011, 2012; Wolfe and Tonsor 2014). Because the collection locations were precisely geo-referenced, we obtained accurate local climate data from geo-referenced climate databases. In addition, these populations appear to share common ancestry. Their co-ancestry results from dispersal across the landscape of northern Spain after emergence from a glacial refugium (Pico *et al*. 2008). These natural populations, therefore, allow the exploration of a series of important ecological and evolutionary questions regarding adaptive divergence from common ancestry. These local populations have evolved functional differences that appear to adapt them to local climates. Traits associated with life history, such as biomass allocation, fecundity, developmental time and demographic traits, such as seed dormancy and timing of seed germination, showed clinal variation along the elevation gradient (Montesinos-Navarro *et al*. 2009, 2011, 2012). Low-elevation populations evolved an early flowering strategy that adapts them to spring heat and drought (Wolfe and Tonsor 2014). These populations also showed variation in genetic diversity, with high-elevation populations being more genetically diverse than low-elevation populations (Gomaa *et al*. 2011). In this study, we address local population evolutionary adaptive divergence in heat shock response and Hsps across a climate gradient. Our study examines mechanisms that may underpin adaptive variation and contributes to growing knowledge on abiotic stress responses in natural plant populations.

In this study, we characterize genetically based differences among 16 wild-collected populations in their responses to heat challenges, focussing on evolutionary divergence in thermotolerance (survival and post-stress growth) and Hsp101 expression.

We addressed the following hypotheses:

- Hypothesis 1: Populations of *A. thaliana* vary in their responses to high temperature when tested in experimentally controlled heat treatments;
- Hypothesis 2: Hsp101 expression variation is positively related with observed variation in thermotolerance;
- Hypothesis 3: The observed variation in thermotolerance and Hsp101 expression is associated with the climate of population origin and is therefore adaptive.

## Methods

### Source populations

Seeds of *A. thaliana* were field-collected and subjected to at least two rounds of single seed descent in controlled environment chambers. Sixty-four genotypes, 4 from each of 16 population locations (Table 1) [also **see Supporting Information—Fig. S1**], were chosen for study. The original seed was collected in collaboration with F. Xavier Picó (Montesinos-Navarro *et al*. 2009, 2011). Dr Picó states that no specific permissions were necessary for any of the 16 collection sites (F. Xavier Picó, pers. comm. to S.J.T., 30 June 2014). The population locations are listed in Table 1. All genotypes have been donated to the *Arabidopsis* Biological Resource Center at Ohio State University (stock CS78884).

**Table 1.** Population locations and climate PCs. Condensed from Tables S1 and S3 in Wolfe and Tonsor (2014).

Population	Longitude	Latitude	Elevation (m a.s.l.)	ClimatePC1	ClimatePC2
PIN	2.6591	41.6592	109	−3.6458	−1.7597
RAB	3.0537	42.3781	110	−4.4803	0.8848
SPE	2.9162	41.9275	332	−2.9213	−1.6334
BAR	2.1278	41.4322	340	−4.58	−1.1973
HOR	2.6202	41.6645	351	−2.7083	−1.8691
ARB	2.4941	41.8143	440	−2.1884	−0.6771
COC	3.1653	42.3263	519	−3.2761	−0.087
POB	1.0235	41.3526	597	−2.0028	3.7342
BOS	0.6913	42.7836	719	2.6056	2.6257
MUR	2.0002	41.6757	836	−2.0033	0.2208
VDM	1.0249	42.0292	912	1.2933	2.6184
ALE	1.3187	42.4105	1163	2.3143	1.2497
BIS	0.5334	42.4878	1397	5.8196	−2.246
PAL	1.2926	42.4312	1491	4.2579	0.6184
VIE	0.7606	42.6256	1538	5.7257	−1.1941
PAN	−0.231	42.762	1664	5.7899	−1.2883

For all collections, we quantified Hsp101 expression directly after heat stress (detailed below). In contrast, we quantified thermotolerance 1 week after heat stress. To obtain these disparate data, we planted two full sets of seeds/populations for each of the four treatments, one destined for Hsp101 quantification immediately following treatment, and the other was grown for 1 week following heat treatment and assayed for seedling survival and root growth.

### Planting designs

Our hypotheses are focussed mainly on understanding population-level differences. We maximized the accuracy of our population mean estimates within the constraints of total size of the experiment. Estimates of individual line performance were less important, especially given the relatively low within-population genetic variance in this highly selfing species.

#### Thermotolerance assay planting design

Four complete replicate sets of agar plates were prepared. Each set included two seedlings of each of the 64 Spanish lines, 128 seedlings total. This gave us eight replicate measures of each population's characteristics. Six seeds were planted per plate; the 128 seeds of a set were distributed across 22 plates. Seeds were randomly assigned to plates, but no plate contained two seeds from any one genotype or population. Because the design does not completely fill 6 × 22 = 132 locations, we were able to also include seeds for a pilot study that are not included in this study in some randomly chosen locations. These pilot study seeds will not be mentioned further.

#### Hsp101 assay planting design

Population-level estimates of Hsp101 expression were based on a single randomly chosen genotype from each population. This was justified because *A. thaliana* is highly selfing (Abbott and Gomes 1989; Nordborg *et al*. 2002) and highly selfing populations tend towards low amounts of internal genetic variation (Loveless and Hamrick 1984; Hamrick and Godt 1996; Duminil *et al.* 2007 but see Platt *et al*. 2010). Each genotype was represented by two replicate plantings, each containing 15–20 seeds; four genotypes were randomly assigned to a plate. This number of seedlings assured sufficient tissue for Hsp101 extraction and quantification.

### Heat stress treatments

The most informative heat stress temperature was unknown at the start of the experiment. Virtually all prior heat stress experiments with *A. thaliana* have been conducted on mid-northern European genetic lines. However, Tonsor *et al*. demonstrated a cline in Hsp101 expression with latitude, suggesting that populations will differ in the temperatures that represent a heat stress (Tonsor *et al*. 2008). Our study populations are from southern European latitudes (Montesinos-Navarro *et al*. 2009). Temperatures in their sites of origin range from cooler to warmer, unlike temperatures in sites of the standard laboratory lines. Given the unknown nature of temperature responses among our lines, we, therefore, imposed two heat challenges, 42 and 45 °C. Our approach was to heat stress 10-day-old seedlings grown on agar plates, and then measure survival, post-stress root growth and quantify Hsp101 expression.

*Arabidopsis* seedlings are known to acquire greater heat tolerance with acclimation. The standard acclimation treatment (AT) in prior published studies was 3 h at 38 °C (Hong *et al*. 2003). We, therefore, compared responses at both 42 and 45 °C for two sets of replicates: those with 38 °C (AT) and thermally naive control treatment (CT) seedlings maintained at 22 °C prior to heat stress. Thus, we employed four thermal treatments. Each of these seedlings were grown at 22 °C prior to and following any heat treatments. Our four thermal treatments were (i) CT42: 42 °C for 3 h; (ii) CT45: 45 °C for 3 h; (iii) AT42: 3 h at 38 °C, recovery at 22 °C for 3 h, then 3 h at 42 °C; (iv) AT45: 3 h at 38 °C, recovery at 22 °C for 3 h, then 3 h at 45 °C.

### Growth protocols and heat stress assays

Seeds were surface-sterilized by exposure to chlorine gas for 3 h. Seeds were grown on gridded square agar plates containing Mirashige and Skoog nutrient solution. For the ‘thermotolerance assay’, seeds were placed on the top grid line and plates were vertically oriented to allow measurement of root growth. For the ‘Hsp101 assay’, the plates were divided into four quadrats and seeds of a randomly chosen genotype were placed in the middle of one of the four quadrats. Plates were oriented horizontally. For all plates in both planting designs, after 5 days of stratification at 4 °C, plates were placed under fluorescent lights and maintained at ∼22 °C with 16 h light, 8 h dark, except during any of the experimental heat treatments. After seed placement, all plates were sealed for the duration of the experiment. Fifteen days after planting, 10 days after emerging from stratification, each replicate heat stress set was exposed to one of the four heat stress treatments as mentioned above. The plates were vertically positioned on racks in a large forced-air drying oven to guarantee rapid air movement and conductive heat transfer. Although we did not measure the temperature of individual plates, heat transfer in the oven is very fast and even. In combination with the low mass of the agar plates, we are certain that plate and agar temperature equilibrated with oven air temperature within a few minutes of placement in the oven. Those sets intended for the thermotolerance assay were returned to 22 °C with 16 h light, 8 h dark and grown for 1 week after heat stress. The sets of plates destined for Hsp101 quantification were harvested immediately after heat stress.

### Thermotolerance measures

We measured seedling survival and post-stress root growth, two widely used measures of heat stress effects (Yeh *et al*. 2012). A seedling was counted as dead if all the leaves turned from green to white. Per cent seedling survival was calculated as the percentage of living seedlings 1-week post-stress for each population. Plates were photographed just prior to heat stress and 1-week post-treatment. Root length was traced using NeuronJ (a macro in NIH ImageJ) for roots in the pre- and post-treatment photos. For each seedling, the post-stress root growth was quantified as the length difference between the pre- and post-treatment images.

### Hsp101 quantification

All seedlings used in the Hsp101 assay were collected immediately after heat treatment and were used for western blot Hsp101 quantification. Seedlings were collected into microcentrifuge tubes and immediately put into liquid nitrogen. A bulk Hsp101 expression standard was prepared by combining multiple leaves from a variety of genotypes that had been subjected to a 42 °C heat treatment. This bulk Hsp101 expression standard was used for comparison across individual gels to minimize gel-to-gel variation. Hsp101 accumulation level was quantified through image analysis of western blots following the procedure of Tonsor *et al*. (2008). We used N-terminal Hsp101 primary antibody from rabbit (Agrisera, AS07253) as the primary antibody and anti-rabbit antibody as the secondary antibody to capture Hsp101.

### Climate quantification

Wolfe and Tonsor combined temperature and precipitation data at the 16 collection sites from the BIOCLIM data set (Hijmans *et al*. 2005, data available at http://www.worldclim.org) and used principal component (PC) analysis to reduce the dimensionality of this data set (Wolfe and Tonsor 2014). Two PCs were significant based on permutation tests. Their ClimatePC1 explained 75 % of the multivariate variance across the 19 BIOCLIM variables, while ClimatePC2 explained 17 % (see Fig. S3 in Wolfe and Tonsor 2014). ClimatePC1 was most strongly associated with temperature and precipitation (cool and moist vs. warm and dry), while ClimatePC2 was associated most strongly with seasonality. We used ClimatePC1 and ClimatePC2 to test hypotheses that climate of origin is associated with variation in both thermotolerance and Hsp101 expression.

### Statistical analyses

One analysis was used for general characterization of responses to heat treatments and to test the hypothesis that populations differed in their responses to the heat treatments. The four heat treatments compose a factorial design in which there are two levels of acclimation (22 °C control or 38 °C acclimation) and two levels of heat challenge (42 or 45 °C). We classified all seedlings as to population of origin, survival or death and post-stress root growth. For all the tests of treatment and population effects on seedling survival described below, logistic regression analysis was applied using Proc GENMOD in SAS (SAS version 9.1, SAS Institute Copyright © 2005). Our logistic regression model, therefore, predicted seedling survival as a function of population, acclimation and heat challenge. Three population interaction effects were also included: population of origin × AT, population of origin × heat challenge and acclimation × heat challenges as well as the three-way interaction of population of origin × AT × heat challenge. Population, AT and heat challenge were all considered as fixed effects in this study. For testing heat treatment effects on post-stress root growth and Hsp101 expression, ANOVAs tested the effects of population, acclimation, heat challenge and their interactions in Proc GLM (SAS version 9.1, SAS Institute Copyright © 2005). In this case, we accounted for the effect of initial size on post-stress growth by using pre-stress root length as a covariate. Both pre- and post-stress root length were log transformed, achieving very close fits to normal distributions of residuals. When interaction tests were clearly non-significant, the analysis was rerun without them. The full-model ANOVA was followed by separate tests by 42 vs. 45 °C heat challenge. For all three measures, analyses were further dissected to examine within-treatment effect of population and its interactions **[see** **Supporting Information—Table S1****]**; however, the power of the within-treatment tests is relatively low. All means comparisons conducted in Proc GENMOND or Proc GLM were followed by Bonferroni critical *P*-value adjustment including Holm's correction (Holland and Copenhaver 1987).

We tested for a relationship between Hsp101 expression and thermotolerance by conducting two regressions. Each regression treated Hsp101 expression (log transformed) as the putative causal variable. One regression tested for Hsp101 expression effects on population mean per cent survival, and one tested for Hsp101 expression effects on population mean post-stress root growth (log transformed). Data were pooled across treatments in these regressions. The regressions were conducted in Proc REG (SAS version 9.1, SAS Institute Copyright © 2005).

We further tested whether variation in thermotolerance and Hsp101 expression matched with variation in the climates of population origin. The climates of population origin, quantified as ClimatePC1 and ClimatePC2 values, were used to examine the evolved effect of past climate on both the population mean thermotolerance measures (per cent seedling survival and post-stress root growth) and on Hsp101 expression. Logistic regression was performed for testing the prediction of post-stress per cent seedling survival with ClimatePC1 and ClimatePC2 using Proc GENMOD (SAS version 9.1, SAS Institute Copyright © 2005). Generalized linear regression using Proc GLM (SAS version 9.1, SAS Institute Copyright © 2005) tested the prediction of past climate influences on post-stress root growth and Hsp101 expression separately. In all cases, the design effects (AT and heat challenge) were treated as fixed effects. The full-model regressions were followed by separate tests of 42 and 45 °C heat challenge.

## Results

### Thermotolerance phenotype depends on acclimation and heat challenge temperature

**Figure 1.**
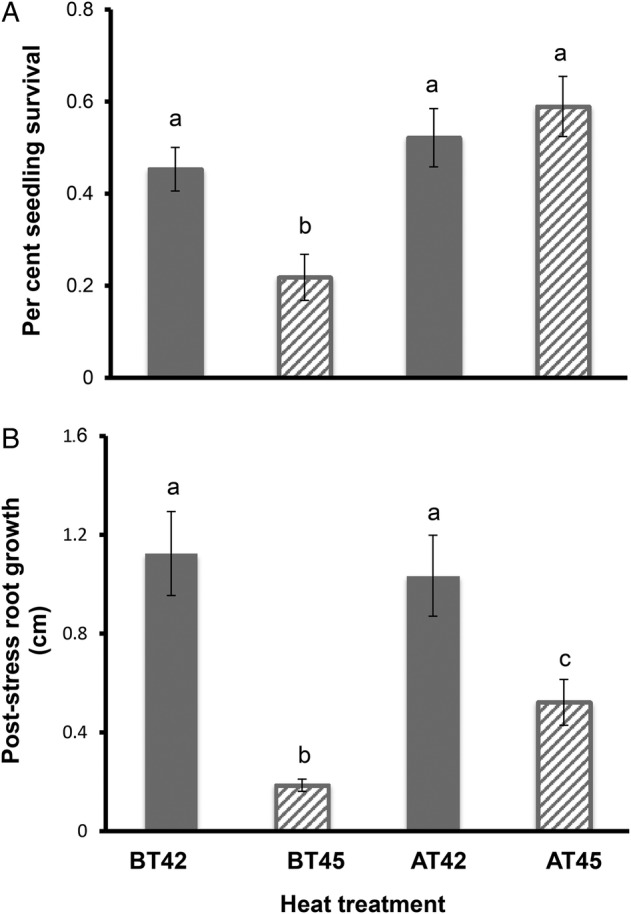
(A) Per cent seedling survival and (B) post-stress root growth 1 week after heat stress, by heat treatment. For per cent seedling survival (A), data displayed are treatment means based on population survival percentage within treatment. For post-stress root growth (B), data displayed are means of individuals within treatments. Vertical lines represent standard errors. Treatments with different letters above the bar are significantly different with an initial rejection criterion of *P* < 0.05 adjusted for multiple comparisons using the sequential Bonferroni method.

ANOVA provided the confidence behind the statements that follow in this paragraph; relevant here are the Heat Challenge and Acclimation × Heat Challenge rows in Table 2a, in conjunction with the associated means comparisons shown in Fig. 1. These analyses indicate that *A. thaliana* accessions from northeastern Spain suffered reduced survival and root growth when exposed directly to 45 °C (CT45 treatments), in keeping with prior experiments using standard laboratory lines of *A. thaliana* (e.g. Queitsch *et al*. 2000). Seedling survival and post-stress root growth differed between the 42 and 45 °C heat challenges (Fig. 1). This difference in heat challenge effect is further separable by the presence or absence of the 38 °C AT. Without acclimation, a 45 °C heat challenge (CT45) significantly decreased per cent seedling survival and post-stress root growth compared with a 42 °C heat challenge (BT42). However, with a 38 °C acclimation, a 45 °C heat challenge (AT45) had no discernable effect on seedling survival, but did show decreased root growth, both compared with the effects of a 42 °C heat challenge (AT42). The effect of a 38 °C AT varies according to response trait and heat challenge. At 42 °C, no effect of acclimation was detected for either response traits (CT42 vs. AT42). However, at 45 °C, acclimation improved thermotolerance for both traits (CT45 vs. AT45). Prior acclimation at 45 °C (AT45) significantly increased root growth by ∼3-fold compared with CT45. However, this amount of growth is still only ∼50 % of the total root length achieved in the CT42 treatment. Thus, at 45 °C, the induction of AT mechanisms is sufficient to insure high survival, but not sufficient for rapid post-stress growth recovery.

**Table 2.** ANOVA table for seedling survival, post-stress root growth and Hsp101 expression in full model and separate analysis by heat challenge temperature. Post-stress root growth was adjusted using pre-stress root growth as a covariate (*P* < 0.0001). —, not applicable. All models exhibit overall significance. When interaction terms were non-significant, the model was run again without the non-significant interaction terms (NS). All reported *P*-values are from the reanalysis.

Source	Seedling survival	Post-stress root growth	Hsp101 expression
(a) Full model			
Model *r*^2^	—	0.89	0.50
Population	0.0007	<0.0001	0.02
Acclimation	<0.0001	0.002	<0.0001
Heat challenge	0.08	<0.0001	<0.0001
Acclimation × Heat challenge	0.0006	0.03	<0.0001
Population × Acclimation	NS	0.09	NS
Population × Heat challenge	0.009	<0.0001	NS
Population × Acclimation × Heat challenge	NS	0.0002	NS
(b) 42 °C heat stress			
Model *r*^2^	—	0.89	0.36
Population	0.01	<0.0001	0.03
Acclimation	NS	NS	0.0004
Population × Acclimation	NS	0.005	NS
(c) 45 °C heat stress			
Model *r*^2^	—	0.94	0.60
Population	0.0005	<0.0001	NS
Acclimation	<0.0001	<0.0001	<0.0001
Population × Acclimation	<0.0001	0.0002	NS

### Hsp101 expression varies with acclimation and heat challenge temperature

**Figure 2.**
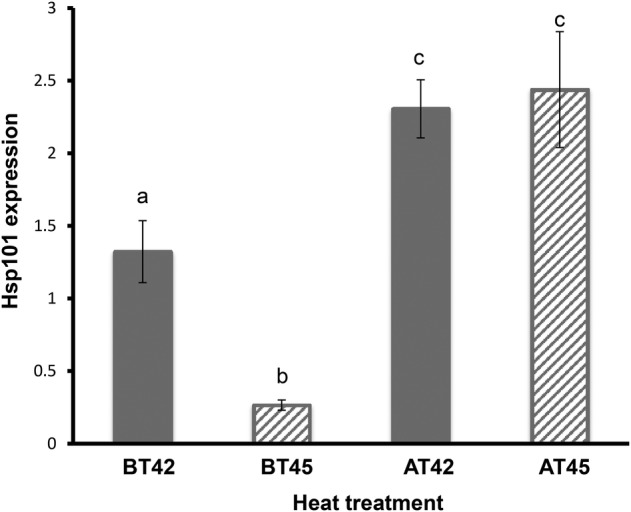
Hsp101 expression by heat treatment. Data displayed are means of individuals within treatments (vertical lines represent standard errors). Hsp101 expression is adjusted to a bulk Hsp101 expression standard to provide a standardized relative comparison among samples. Treatments with different letters above the bar are significantly different with an initial rejection criterion of *P* < 0.05 adjusted for multiple comparisons using the sequential Bonferroni method.

**Figure 3.**
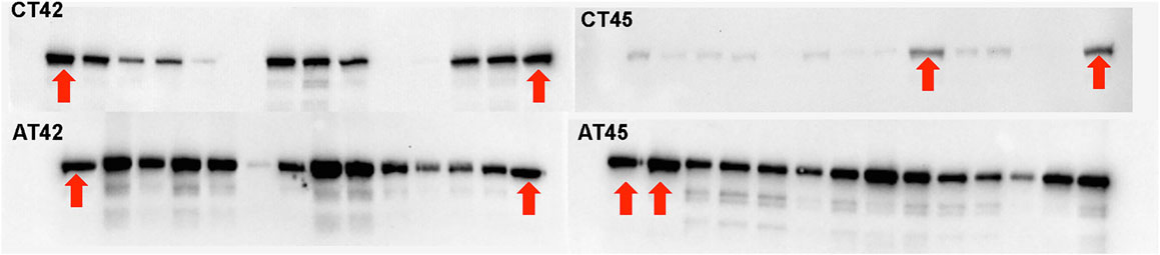
Western blots showing differences in Hsp101 expression among treatments. Variation within treatments is due to significant population variation in expression as well as sample variation in total protein content. A molecular ladder was used in each gel to identify Hsp101 position (ladder not visible in images). Within treatment, genotypes within populations were randomly assigned to the lanes of western blots. For analysis, variation in the amount of protein loaded was used to adjust Hsp101 expression per unit protein. A bulk sample was created and loaded in two lanes on each gel as a control for gel-level variation. Arrows indicate the bulk controls in each western blot.

Hsp101 expression was significantly up-regulated in acclimated plants that were later exposed to both 42 and 45 °C (Figs 2 and 3; Table 2; *P* < 0.0001). The content of Hsp101 as a result of acclimation at AT42 and AT45 was indistinguishable (Figs 2 and 3). The heat challenges also led to an increase in Hsp101 expression at both CT42 and CT45 (i.e. without prior acclimation). However, the amount of Hsp101 expressed at CT42 and CT45 was significantly less than that observed at AT42 and AT45: 34 and 85 % less Hsp101 at CT42 (Table 2b; Fig. 2; *P* = 0.0004) and CT45, respectively (Table 2c; Fig. 2; *P* < 0.0001). This difference in Hsp101 expression between the two CT treatments may be caused by strongly compromised cellular function at 45 °C and only partially compromised cellular function at 42 °C. Yet even this reduced expression level at 42 °C was sufficient to enhance survival and post-stress root growth to an extent that was indistinguishable from that in seedlings in the AT42 treatment. However, the same was not true for CT45 plants; those plants had greatly reduced Hsp101 expression (85 % less) compared with AT45 plants, average seedling survival rates of only 40 % and post-stress root growth of only ∼20 % of that observed at AT45. Our interpretation is that 42 °C heat challenge is mild enough that many plants can maintain sufficient cellular function to rapidly induce acquired thermotolerance mechanisms, including Hsps. In contrast, at 45 °C, cellular function appears to be compromised so quickly that induction of thermotolerance mechanisms is substantially impaired and the seedlings cannot protect cellular function from further damage. The differences among populations that we see in Hsp101 accumulation in acquired thermotolerance may be due in part to evolved differences in the maximum level accumulation of Hsp101. It may also be due to the differential response to 38 °C among these populations, that is, some populations may produce substantially more Hsp101, while some may produce less at 38 °C. Although we do not have a way to address this, the difference in Hsp101 response to the 38 °C acclimation temperature is very unlikely to contribute to the difference we see. Additionally, 42 °C might not represent a significant stress, while 45 °C represent a more stressful temperature.

### Populations of *A. thaliana* vary in their responses to high temperature

Overall, we observed genetically based variation among populations in thermotolerance (seedling survival: *P* = 0.0007; post-stress root growth: *P* < 0.0001; Table 2a) and Hsp101 expression (*P* = 0.02; Table 2a). Within heat treatments, populations differ significantly except in seedling survival at CT42 **[see** **Supporting Information—Table S1****]**. Populations significantly vary in post-stress root growth within all four treatments **[see** **Supporting Information—Table S1****]**. Moreover, populations also differ significantly in their responses to the two heat challenges, as reflected in the Population × Heat challenge interaction (Table 2a), indicating complex among-population variation in stress-dependent responses.

Significant variation in Hsp101 expression was observed among populations when all the data were pooled (Table 2a; Fig. 3). However, the inherent variability in Hsp101 expression and in quantification from western blots, together with limited sample number, resulted in low power to detect differences among populations when treatments were analysed separately **[see** **Supporting Information—Table S1****]**. Thus, in further analyses and discussion, we focus on the general relationship with the pooled data between Hsp101 expression and the thermotolerance measures at the population level.

### Variation in Hsp101 accumulation is positively associated with variation in thermotolerance

**Figure 4.**
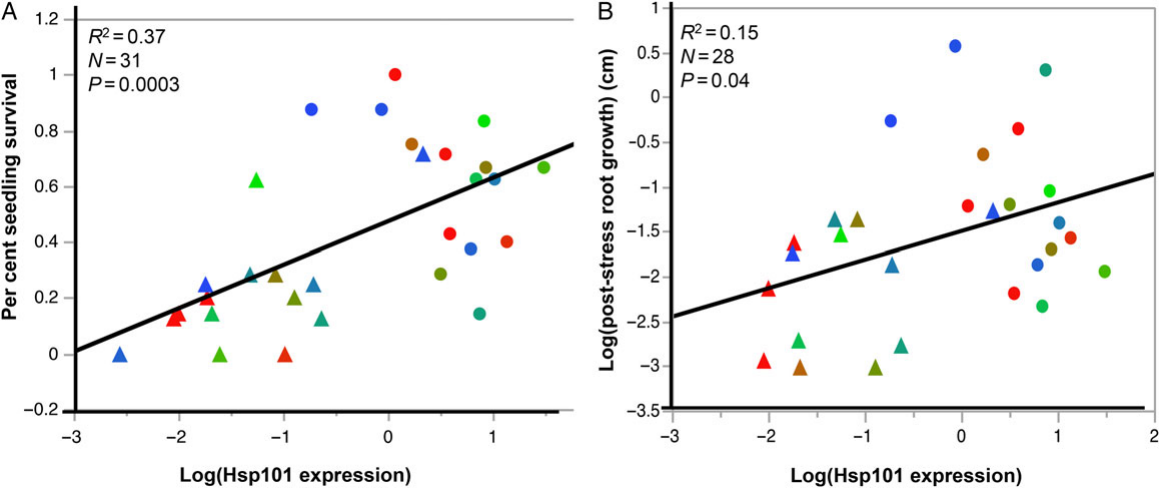
Regression of (A) per cent seedling survival and (B) post-stress root growth (cm, log transformed) on Hsp101 expression (log transformed) at 45 °C heat challenge (BT45 and AT45). Data displayed are population means of each treatment for all four treatments. (A) Per cent seedling survival = 0.47 + 0.16 × log (Hsp101 expression); (B) log (post-stress root growth) = −1.50 + 0.32 × log (Hsp101 expression). Triangle: BT45; circle: AT45. The colours of the populations were arranged along a red–green–blue gradient, from low to high elevation, e.g. red represents low-elevation populations, while blue represents high-elevation populations.

At 45 °C, higher Hsp101 accumulation was associated with higher per cent seedling survival, explaining 37 % of the variation (Fig. 4A; *P* = 0.0003). Similarly, higher Hsp101 accumulation was associated with increased post-stress root growth, accounting for 15 % of the variation (Fig. 4B; *P* = 0.04). Plants with low Hsp101 accumulation exhibited very low rates of survival and below average rates of post-stress root growth. In contrast, Fig. 4 also shows that plants with high Hsp101 accumulation range from very low to very high survival and post-stress root growth. This pattern demonstrates that Hsp101 up-regulation is necessary but not sufficient to ensure high levels of thermotolerance.

It is important to note that many other Hsps are co-regulated with Hsp101. Thus, we cannot ascribe a direct causal relationship to the significant regression of survival or post-stress growth on Hsp101 content. In fact, it is most likely that thermotolerance is conferred by a whole suite of genes, with Hsp101 playing a direct and essential role (Hong *et al*. 2003).

### The observed variation in thermotolerance and Hsp101 expression appears to be adaptive

Both thermotolerance measures and Hsp101 accumulation co-varied with ClimatePC1 of the populations' sites of origin under some but not all treatments (Table 3). Overall, ClimatePC1 significantly predicted seedling survival (Table 3a; *P* = 0.04). That is, populations from the cooler, moister end of the climate gradient exhibited increased per cent seedling survival compared with populations from hotter and drier climates. This relationship was also significant at the 45 °C heat challenge (Table 3c; *P* = 0.008), and this effect at 45 °C appears to have driven the significance of the analysis overall. Regression of post-stress root growth on ClimatePC1 was not significant overall (Table 3a). However, for plants exposed to 45 °C, post-stress root growth increased significantly with ClimatePC1 (i.e. increased towards the cooler and moister end of the climate gradient compared with the hotter and drier end; Table 3c; *P* = 0.0003). Hsp101 expression was not significantly associated with ClimatePC1 overall (Table 3a) but was positively associated with ClimatePC1 at 42 °C (Table 3b; *P* = 0.01). ClimatePC2 was not a significant predictor of thermotolerance measures or Hsp101 accumulation in either the full model or in the separate treatment analyses (Table 3).

**Table 3.** ANOVA table for seedling survival, post-stress root growth and Hsp101 expression with ClimatePCs in full model and in separate analyses by heat challenge temperature. Post-stress root growth was adjusted using pre-stress root growth as a covariate (*P* < 0.0001). —, not applicable. All models are significant. All reported *P*-values are from a reanalysed model after removing the non-significant interaction terms (NS).

Source	Seedling survival	Post-stress root growth	Hsp101 expression
(a) Full model			
Model *r*^2^	—	0.86	0.41
ClimatePC1	0.04	NS	NS
ClimatePC2	NS	NS	NS
Acclimation	<0.0001	0.001	<0.0001
Heat challenge	0.09	<0.0001	<0.0001
Acclimation × Heat challenge	0.001	0.04	<0.0001
(b) 42 °C heat stress			
Model *r*^2^	—	0.83	0.17
ClimatePC1	NS	NS	0.01
ClimatePC2	NS	NS	NS
Acclimation	NS	NS	0.001
(c) 45 °C heat stress			
Model *r*^2^	—	0.92	0.49
ClimatePC1	0.008	0.0003	NS
ClimatePC2	NS	NS	NS
Acclimation	<0.0001	<0.0001	<0.0001
ClimatePC1 × Acclimation	0.02	NS	NS

## Discussion

This study demonstrated a general pattern among plant populations of natural variation in thermotolerance, here measured by seeding survival and post-stress root growth, and accompanied by increases in accumulation of Hsp. Furthermore, the pattern of variation in thermotolerance is associated with variation in the climates of the populations' sites in northeastern Spain. We found that pre-exposure to mild heat stress resulted in acquired thermotolerance and an associated up-regulation of Hsp101. The AT increased thermotolerance substantially. Hsp101 expression also varies among populations and shows the first evidence of genetically based variation in expression associated with climate.

It may seem counter-intuitive that the genetic lines from cooler, moister end of the climate gradient exhibited higher seedling survival and post-stress root growth than lines from the hotter, dryer end of the gradient. The lines from the cooler end of the gradient also showed greater accumulation of Hsp101. Importantly, these results are concordant with results of a previous study of geographic variation in Hsp101 expression (Tonsor *et al*. 2008). Genetic lines of *A. thaliana* from cooler, moister, northern latitudes exhibit greater Hsp101 expression than lines from the southern limits of the species' range (Tonsor *et al*. 2008). Populations of *Arabidopsis* appear to be differentiated in their mechanisms of response to heat stress with more southern populations relying less on acquired thermotolerance. Previous studies in the same populations used here suggest that low-elevation populations may instead avoid heat by maturing earlier (Montesinos-Navarro *et al*. 2012; Wolfe and Tonsor 2014). A similar pattern, which populations from cooler environments had higher induced/acquired thermotolerance, was also found by Barua *et al*. (2008). A number of other factors may also contribute to the seemly counter-intuitive results. Firstly, populations from higher altitudes and cooler climate are exposed to more variable environments—higher range of temperatures, such as high variability in annual or diurnal temperature. Barua *et al*. (2008) further found that variation in induced tolerance and Hsps was related to temperature variability, in that populations that are more likely to experience multiple heat stresses had higher tolerance and Hsp accumulation. Further analysis on the microclimate variability will provide more information on these populations' local climate conditions. Secondly, plants from lower altitudes and warmer climate may rely on basal mechanisms of thermotolerance that are thought to be lower cost compared with induced mechanisms. Thirdly, plants from lower altitudes and warmer climate may simply avoid heat stress by maturing earlier. Finally, plants from high altitudes are faced with other stresses like UV-B radiation, which may be correlated with thermotolerance and Hsps. However, our populations are not sufficiently high in altitude that annual mean UV is meaningfully greater along the elevation gradient (data extracted from DIVA-GIS 7.5.0).

The seeds used in this experiment are descended from field-collected genotypes through at least two, most of them three or four, generations of single seed descent in growth chambers. Within the growth chambers, individual seed plants were randomized in their locations. We can, therefore, be confident that the differences observed among populations reflect differences in population genetic composition rather than uncontrolled environmental differences during the experiment or environment maternal effects carried over from the field. Pico *et al*. (2008) demonstrated that the populations from this region of Spain share a common ancestry, no doubt resulting from their ancestor's emergence from their ice age refugium and their subsequent spread across the landscape of northeastern Spain (Pico *et al*. 2008). The significant association of seedling survival rates with the first PC of climate variation (ClimatePC1) suggests the signature of adaptation to the specific habitats from which the populations were collected. That we only detect an association of variation in seedling survival, post-heat root growth or Hsp101 expression with climate of origin under some circumstances suggests three additional explanations for the observed population variation. First, the power of this experiment may not have been sufficient, given the inherent variability in seedling root growth and Hsp101 quantification in our experimental design and execution. Second, macro-scale climate may not be the only factor involved in adaptation. In the highly dissected topography of northeastern Spain, many unmeasured site characteristics can influence microclimate, including slope, aspect, height of surrounding vegetation and thermal properties of the substrate. All of these could either directly influence the temperatures experienced or influence the trade-offs involved in implementing various potential thermotolerance mechanisms. Finally, founder effect and random genetic drift may account for some of the observed variations. However, the adaptive clines observed in a great many traits previously examined in these populations make it unlikely that random genetic drift is the predominant cause of the observed variation (Montesinos-Navarro *et al*. 2011, 2012; Wolfe and Tonsor 2014).

Nearly all of the existing knowledge of thermotolerance and heat shock response in *A. thaliana* has been gained from studies of seedlings. It is important to note that these studies cannot fully put cellular mechanisms of thermotolerance into the context of mechanisms of thermotolerance relevant in field settings. First, the seedling stage is the least likely stage to experience lethally high temperatures. *Arabidopsis thaliana* seeds germinate under the most benign conditions experienced by the plant—the cool, moist periods of late fall and early spring. Second, like most plants, *A. thaliana* possesses a number of mechanisms for avoiding high temperatures that cannot be used at the seedling stage because the necessary structures and functions simply are not present. These include avoidance of periods of high temperature through the evolution of altered life-history timing (Montesinos-Navarro *et al*. 2012), variation in constitutive high leaf angle, leaf hyponasty and petiole elongation in response to elevated temperature (Gray *et al*. 1998; Polko *et al*. 2011) and transpirational cooling. Full understanding of high-temperature responses and their consequences will require integrated study of cellular, physiological and developmental responses throughout the plant life cycle.

## Conclusions

This study focuses on identifying the adaptive variation in response to high-temperature stress across an elevation gradient in natural *A. thaliana* populations. We show that the accumulation of Hsp101, an important Hsp known to be essential for acquired thermotolerance, was positively associated with seedling survival and post-stress root growth. Pre-acclimation significantly increased thermotolerance at 45 °C but not at 42 °C. Furthermore, both Hsp101 and thermotolerance were correlated with the climate variation of home sites. Our study contributes to growing knowledge on abiotic stress responses in natural plant populations.

## Sources of Funding

Funding was provided by US National Science Foundation Grant IOS-1120383 to S.J.T. and a University of Pittsburgh Dietrich College of Arts and Sciences First Year Fellowship to N.Z.

## Contributions by the Authors

N.Z. and S.J.T. designed the experiment. N.Z., B.B. and J.R. performed the experimental work. N.Z. and S.J.T. performed analyses. All co-authors discussed and interpreted results prior to writing. N.Z. and S.J.T. wrote the manuscript.

## Conflict of Interest Statement

None declared.

## Supporting Information

The following additional information is available in the online version of this article –

**Table S1.** Significance values for tests of within-population variation in seedling survival, post-stress root growth and Hsp101 expression. Each treatment was tested in a separate analysis.

**Figure S1.** Geographic location of the 16 populations used in this study (from Fig. 1, Montesinos-Navarro *et al*. 2011).

Additional Information
