# Supplementary material for: Natural populations of Arabidopsis thaliana differ in seedling responses to high-temperature stress
Source: AoB Plants. 2015 Aug 18;7:plv101. doi: 10.1093/aobpla/plv101 (PMC4598537; doi:10.1093/aobpla/plv101)
Supplement: Additional Information [file supp_7_plv101_index.html]

Natural populations of Arabidopsis thaliana differ in seedling responses to high-temperature stress — Additional Information 

# Natural populations of *Arabidopsis thaliana* differ in seedling responses to high-temperature stress

## Additional Information

Additional Information

- Supplementary Table 1 - docx file
- Supplementary Figure 1 - docx file
