# Supplementary material for: Natural populations of Arabidopsis thaliana differ in seedling responses to high-temperature stress
Source: AoB Plants. 2015 Aug 18;7:plv101. doi: 10.1093/aobpla/plv101 (PMC4598537; doi:10.1093/aobpla/plv101)
Supplement: Additional Information [file supp_plv101_plv101supp_table1.docx]

Table S1. Significance values for tests of within population variation in seedling survival, post-stress root growth and Hsp101 expression.

Each treatment was tested in a separate analysis.

C

C

CT42: 42°C for 3hrs; CT45: 45°C for 3hrs; AT42: 3hrs at 38°C, recovery at 22°C for 3hrs, then 3hrs at 42°C; AT45: 3hrs at 38°C, recovery at 22°C for 3hrs, then 3hrs at 45°C.
