# Supplementary material for: Natural populations of Arabidopsis thaliana differ in seedling responses to high-temperature stress
Source: AoB Plants. 2015 Aug 18;7:plv101. doi: 10.1093/aobpla/plv101 (PMC4598537; doi:10.1093/aobpla/plv101)
Supplement: Additional Information [file supp_plv101_plv101supp_fig1.docx]

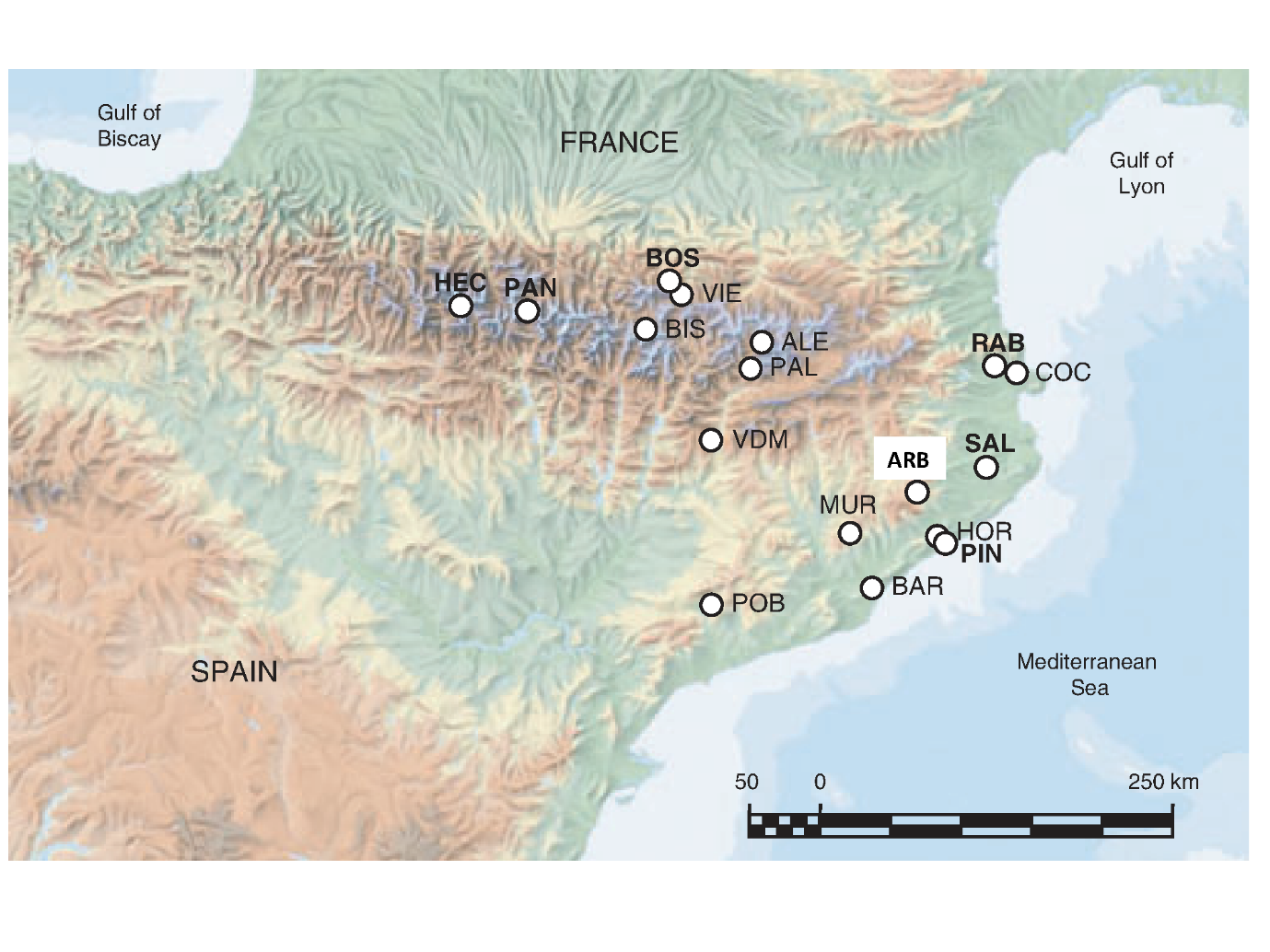


Fig. S1 Geographic location of the 16 populations used in this study (From Fig.1, Montesinos-Navarro *et al* 2011).
